# Reconciliation (Poetry)

**Published:** 1881-12

**Authors:** Caroline A. Mason


### RECONCILIATION.

If thou wert lying, cold and still and white,
In death's embrace, O mine enemy !
I think that if I came and looked on thee,
I should forgive ; that something in the sight
Of thy still face would conquer me, by right
Of death's sad impotence, and I should see
How pitiful a thing it is to be
At feud with aught that's mortal.

So, to-night
My soul, unfurling her white flag of peace—
Forestalling that dread hour when we may meet—
The dead face and the living—fain would cry
Across the years, "O, let our warfare cease !
Life is so short, and hatred is not sweet :
Let there be peace between us ere we die."

*—Caroline A. Mason in Scribner.*

